# Pakistan’s path forward in DR-TB management: insights from global implementation of BPaL/BPaLM regimen

**DOI:** 10.1097/MS9.0000000000003548

**Published:** 2025-07-10

**Authors:** Santosh Sah, Areeba Ahsan, Tularam Yadav

**Affiliations:** a Department of Internal Medicine, Dow University of Health Sciences, Karachi, Pakistan; b Primary Healthcare Centre (PHCC) Jhorahat, Koshi, Nepal

## Abstract

Drug-resistant tuberculosis (DR-TB) is a serious public health threat, and Pakistan is one of the most impacted nations. The long treatment duration of traditional regimens puts a great burden on healthcare systems, especially in resource-constrained environments. Accordingly, the World Health Organization launched the Bedaquiline, Pretomanid, and Linezolid (BPaL)/Bedaquiline, Pretomanid, Linezolid, and Moxifloxacin (BPaLM) regimen – a 6-month, all-oral therapy consisting of BPaLM. With stated success rates as high as 90%, these regimens present an exciting alternative to traditional treatments. Yet, their integration into current treatment programs is hindered by policy lags, poor diagnostic infrastructure, and difficulty in maintaining patient compliance. This brief communication explores the promise of BPaL/BPaLM to enhance DR-TB cure rates while pinpointing major hurdles to its use in Pakistan. Enhancing diagnostic capacity, upgrading healthcare infrastructure, and accelerating policy adjustment are crucial steps toward maximizing DR-TB management in high-burden countries.

## Introduction

Tuberculosis (TB), remains a major global health concern, particularly due to the increasing prevalence of drug-resistant TB (DR-TB). This form of TB contributes substantially to morbidity, mortality, and healthcare costs worldwide. The World Health Organization (WHO) categorizes drug-resistant TB into five distinct groups based on drug resistance patterns: Isoniazid-resistant TB, rifampicin-resistant TB (RR-TB), multidrug-resistant TB, pre-extensively drug-resistant TB, and extensively drug-resistant TB (XDR-TB). Accurate detection of these forms requires bacteriological confirmation through rapid molecular diagnostics, culture methods, or sequencing technologies^[1]^.

**Figure 1.**
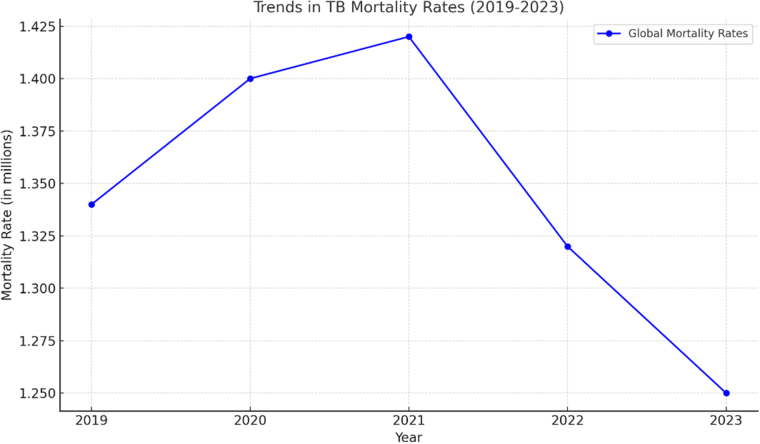
Data sourced from global TB surveillance reports (1). TB mortality rates are presented in millions of deaths per year. The decline in mortality post-2021 is attributed to improved TB diagnostics, treatment accessibility, and healthcare interventions.

Despite advancements in treatment, only 44% of the estimated 400 000 global MDR/RR-TB cases received treatment in 2023, which highlights a considerable gap in care provision. And Pakistan ranked 5th among 10 countries contributing to 75% of the treatment gap for MDR/RR-TB^[2]^. TB caused an estimated 1.25 million deaths in 2023, marking a consistent decline from previous years (1.32 million in 2022, 1.42 million in 2021, 1.40 million in 2020) and falling below pre-pandemic mortality of 1.34 million in 2019^[1]^ demonstrated in Figure 1. To enhance treatment outcomes, the WHO initially recommended a 9–11-month short oral regimen (SSOR) and an 18-month long oral regimen (SLOR) for DR-TB, until it introduced the combined Bedaquiline, Pretomanid, Linezolid, and Moxifloxacin (BPaLM) regimen, as a shorter, all-oral treatment option, which demonstrated a 90% success rate in clinical trials (Nix-TB and ZeNix), surpassing previous regimens^[3–5]^. A retrospective study conducted by Gualano *et al*^[6]^, Rome, Italy, from December 2022 to June 2024 revealed 17 patients out of 19 completed the BPaL/BPaLM regimen, with a treatment success rate of 90% (17/19). While a study in an Asian population (Thailand) documented the efficacy and safety of BPaL/BPaLM, indicating that the sputum culture conversion at 8 weeks for the BPaL regimen was 8/10 (80%) and for the BPaLM regimen was 18/18 (100%)^[7]^.

By the end of 2023, 58 countries had adopted the BPaLM regimen, marking a significant step forward in DR-TB management^[1]^. However, resource-constrained settings like Pakistan continue to face barriers to implementation due to infrastructure and financial limitations. This editorial explores strategies to overcome these challenges, drawing from the global adoption of the BPaLM regimen to strengthen Pakistan’s DR-TB management framework.

### Cost-effectiveness and global outcomes of BPaL/BPaLM

Numerous studies highlight the cost-effectiveness of the 6-month BPaL/BPaLM regimen in DR-TB patients, particularly in high-burden settings^[8–11]^. A study from South Africa, reported that total patient costs for BPaL ($56.6) were significantly lower than for SSOR ($228.1) and SLOR ($224.7), with a 75% reduction driven mainly by decreased non-medical expenses such as transportation, food, nutritional supplements, income lost due to time spent seeking TB care, etc^[8]^.

A similar study in Moldova found that implementation of BPaLM regimen is projected to save Moldova’s national TB budget $7.1 million over a 5-year period^[9]^. In the Philippines, patient costs with BPaL were 37%–50% lower than SSOR/SLOR, while provider savings ranged from 36% to 80% per successful treatment^[10]^. Another comparative study done on four countries (Pakistan, the Philippines, South Africa, and Ukraine) estimated per-patient savings of $478–$2636, with 5-year savings ranging from $14 to $61.6 million depending on each country’s DR-TB burden and the speed of transitioning from current to new regimens^[11]^. Further analysis in South Africa, Georgia, and the Philippines suggests that BPaL/BPaLM is cost-saving compared to standard XDR-TB treatments, reinforcing its economic viability^[12]^ as summarized in Table 1. Therefore, the rapid implementation of BPaL/BPaLM regimens is strongly justified based on economic benefits and improved treatment outcomes. The anticipated cost savings could be reinvested to enhance diagnostic access and treatment support.

**Table 1:** Cost-effectiveness and economic outcomes of BPaL/BPaLM regimens for drug-resistant tuberculosis^[8–12]^

Country/study	Comparison	Cost/outcome measure	Key findings
South Africa^[8]^	BPaL vs. SSOR/SLOR	Patient cost:	75% reduction in patient costs, mainly due to lower non-medical expenses.
		• BPaL = $56.6 SSOR = $228.1 SLOR = $224.7	
Moldova^[9]^	BPaLM vs. existing regimens	Projected national TB budget impact over 5 years	$7.1 million savings with BPaLM implementation.
Philippines^[10]^	BPaL vs. SSOR/SLOR	• Patient cost: 37%–50% lower	Significant savings for both patients and health system.
		• Provider cost savings: 36%–80% per successful treatment	
Multi-country: Pakistan, Philippines, South Africa, Ukraine^[11]^	BPaL/BPaLM vs. country regimens	• Per-patient savings: $478–$2636	Substantial savings across various DR-TB burden settings.
		• 5-year savings: $14–$61.6 million depending on country burden and rollout	
South Africa, Georgia, Philippines^[12]^	BPaL/BPaLM vs. standard XDR-TB treatment	Comparative cost-effectiveness analyses	Proven cost-saving, reinforcing the case for rapid adoption of BPaL/BPaLM.

This table summarizes the key findings from economic evaluations of BPaL/BPaLM regimens implementation across different high-burden settings^[9–12]^.

### Current status and challenges in Pakistan

Among WHO regions, the European Region achieved the highest coverage at 78%, while the South-East Asian Region had the lowest at 39%^[1]^, and Pakistan, one of the highest DR-TB burden countries, failed to implement the new regimen with proper effectiveness. Delays in diagnosis, improper drug regimens, insufficient follow-up, and lack of social support programs still remain major factors contributing to drug resistance in Pakistan. The Association for Social Development (ASD) enrolled 206 participants with drug-resistant tuberculosis (DR-TB) in Pakistan for treatment under a pilot program from October 2022 till February, 2023 using the BPaL/M regimen, and reported that out of 113 treatment outcomes declared, so far 105 (95%) have been successful, and as an outcome of these results, Pakistan has updated its treatment guidelines for DR-TB to enable the use of these 6-month, all-oral regimens^[13]^. ASD, in partnership with Pakistan’s National TB Control Program (NTP) and TB Alliance, has recruited participants at four sites in Punjab and Khyber Pakhtunkhwa provinces: Rawalpindi, Multan, Lahore, and Peshawar, and is further expanding the program. Alongside, ASD supported the rollout by providing technical expertise, developing training materials, adherence guidelines, and performance monitoring tools. TB Alliance has granted Remington Pharmaceutical Industries (Pvt.) Ltd. a royalty-free license to market the anti-tuberculosis medicine Pretomanid, as part of the all-oral, 6-month BPaL/M regimen^[13]^.

According to National TB Control Program 2022 figures, the current estimate of incident TB in Pakistan is 611 000 cases, with an incidence of MDR/RR-TB of 16 000 cases. 2878 DRTB cases were reported in 2021, 3682 in 2022, and 395 in 2023. To combat with the on growing challenges following measures have been taken by the government of Pakistan regulatory authorities^[14]^.

- Treatment centers: 36 (2021) → 43 (2022) → 51 (2023).
- Support for DR-TB patients: PKR 12 000/month incentive to the patient.
- Diagnostics: GeneXpert machines (411 → 774), digital X-ray, and AI (12 → 136).
- Laboratories capacity: 37 divisional labs upgraded with 25 PCR machines.
- Surveys: TB prevalence, patient cost, drug resistance.
- Surveillance: DHIS-2 case-based tracking.
- Collaboration: Enhanced coordination with medical associations, private sector, and international organizations.

### Scaling up in a resource-constrained settings

The primary challenge now lies in rapidly scaling up the use of BPaL/BPaLM regimens. However, its widespread adoption is hindered by several real-world challenges, which are especially pronounced in high-burden, resource-constrained settings where DR-TB care is most critical. The challenges start from a lack of proper diagnostic tools, confirming eligibility for the BPaL/M regimen through drug susceptibility testing, regular monitoring to manage the adverse effects, monitoring the patients’ adherence to the drugs, etc^[10,15]^. Despite its cost-effectiveness in the long term, its upfront costs, combined with the needed infrastructure upgrade, can have a significant strain on national TB program budgets. It needs adequate budget allocation along with proper policy making by the government bodies. Many countries have not adopted this regimen yet because of delayed approvals and policy alignment. While the regimen proves cost-effective over time, the initial procurement costs can be substantial, particularly for countries dependent on donor funding^[8,10]^. Even though BPaL/M is a shorter regime, there is still an unexplained anxiety surrounding it especially in patients that experience any of the serious side effects. These barriers (outlined in Table 2) require strong epidemiologic, economic, and moral frameworks that adequately address the need for support among countries that endeavour to expand.

**Table 2:** Challenges in scaling up BPaL/BPaLM regimens in resource-constrained settings^[8,10,15]^

Challenges	Description	Proposed solutions
Diagnostic limitations	Lack of proper diagnostic tools, including drug susceptibility testing and routine monitoring.	Strengthen diagnostic capacity with rapid molecular tests (GeneXpert, Line Probe Assay).
Adverse effect monitoring	Regular monitoring is needed to manage adverse effects related to the regimen.	Train healthcare workers to manage and monitor side effects effectively.
Patient adherence	Ensuring patients adhere to the treatment regimen, particularly with a shorter treatment course and potential side effects.	Provide support through nutritional assistance, transportation, and counseling services.
Upfront costs and infrastructure	The initial procurement costs and infrastructure upgrades, such as laboratory improvements and skilled technician training, can strain national budgets.	Secure adequate funding, policy alignment, and donor support for initial costs and infrastructure improvements.
Policy and regulatory delays	Delayed approvals and lack of policy alignment can hinder the rapid adoption of BPaL/BPaLM regimens.	Advocate for faster regulatory approvals and policy alignment to enable quick adoption.
Public awareness and acceptance	Anxiety surrounding the new regimen, especially among patients experiencing side effects, may delay acceptance of the treatment.	Launch public awareness campaigns to promote early diagnosis, treatment, and mitigate concerns regarding side effects.
Funding and resource allocation	The substantial upfront costs, especially for countries dependent on donor funding, create challenges in implementing the regimen.	Secure long-term funding through government bodies and international donors.
Sustainability and long-term monitoring	The need for robust research, surveillance systems, and monitoring of long-term outcomes to track treatment efficacy and resistance trends.	Build surveillance systems and invest in ongoing research to improve DR-TB management.

This table outlines the key challenges faced by resource-constrained settings in scaling up BPaL/BPaLM regimens, alongside potential solutions to ensure successful implementation. The challenges include diagnostic limitations, monitoring requirements, patient adherence, and the financial and infrastructural constraints that hinder the widespread adoption of this regimen^[8,10,15]^.

SSOR, short standard oral regimen; SLOR, long standard oral regimen; XDR-TB, extensively drug-resistant tuberculosis; BPaL/BPaLM, bedaquiline, pretomanid, linezolid ± moxifloxacin.

However, to effectively implement this regimen in Pakistan, strengthening diagnostic capacity with rapid molecular tests (GeneXpert, Line Probe Assay) and upgrading healthcare infrastructures like laboratories and skilled technicians are essential. Training healthcare workers to manage side effects and ensuring drug availability, including Pretomanid, are critical. Patient adherence can be improved through nutritional, transportation, and counselling support. Policy alignment and securing funding are needed to cover upfront costs and expedite national integration. Public awareness campaigns should promote early diagnosis and treatment. Additionally, robust research and surveillance systems are vital to monitor long-term outcomes and resistance trends, enabling data-driven improvements in DR-TB management.

### Limitations

Though encouraging, the BPaL/BPaLM regimen also has some challenges. Despite having better efficacy as compared to the current standard of care, it’s not completely side-effect-free. Linezolid, a component of the regimen, is linked to significant side effects. These impedances seek regular laboratory investigations (full blood count) and clinical investigation that are frequently absent in the resource limited settings. Sometimes healthcare practitioners themselves require supervision in rural areas in order to understand and treat such toxicities. And these may overburden the already weak healthcare facilities in Pakistan. Additionally, low awareness, logistical barriers in rural areas, and slow processes of regulatory approvals may undermine efficient interventions.

## Conclusion

The BPaL/BPaLM regimen represents a transformative opportunity to improve DR-TB outcomes, offering a shorter, more effective, and cost-efficient approach. However, its success depends critically on timely government endorsement, broader scope of molecular diagnostics, and consistent support. Achieving affordability through computed government investment and international alliances is crucial. Furthermore, specialized DR-TB units and DR-TB nurses are required to manage adverse effects to patient compliance. A much more consolidated patient-centric approach will be necessary to incorporate these plans and enable Pakistan to effectively combat DR-TB.

## Data Availability

Not available.
